# Modelling the significance of social media marketing activities, brand equity and loyalty to predict consumers’ willingness to pay premium price for portable tech gadgets

**DOI:** 10.1016/j.heliyon.2022.e10145

**Published:** 2022-08-11

**Authors:** Chinnasamy Agamudainambhi Malarvizhi, Abdullah Al Mamun, Sreenivasan Jayashree, Farzana Naznen, Tanvir Abir

**Affiliations:** a Faculty of Management, Multimedia University, 63100, Cyberjaya, Malaysia; b UKM - Graduate School of Business, Universiti Kebangsaan Malaysia, UKM Bangi, Malaysia; c UCSI Graduate Business School, UCSI University, Kuala Lumpur, Malaysia; d Faculty of Business and Entrepreneurship, Daffodil International University, Bangladesh

**Keywords:** Social media marketing activities, Brand awareness, Brand image, Brand loyalty, Willingness to pay premium price

## Abstract

In order to sustain business operations during the COVID-19 pandemic, nearly all industries have to adopt online technology and social media marketing activities (SMMAs). Globally, portable tech gadgets are rapidly expanding, but empirical studies on SMMAs in relation to portable tech gadgets in Malaysia have remained scarce. Therefore, this study examined the elements of SMMAs and their influence on brand equity in terms of brand awareness (BBA) and brand image (BBI) as well as brand loyalty (BRL) and willingness to pay premium price (WPP) among Malaysian consumers of portable tech gadgets users. Five components of SMMAs, namely entertainment (ENT), interactivity (INT), trendiness (TRE), customisation (CUS), and electronic word-of-mouth (EWOM), were examined to understand how SMMAs influence BBA, BBI, BRL, and WPP. An online survey was conducted with 1332 Malaysian youths who used social media platforms maintained by portable tech gadget brands as their marketing strategies. The gathered data were evaluated using structural equation modelling. The study's results indicated the significant and positive effects of TRE, CUS, and EWOM on BBA and BBI. INT was revealed to have no significant impact on BBA and BBI. Furthermore, BBI and BBA partially mediated the relationships of the components of SMMAs with WPP. As for the theoretical underpinning, this study used the stimulus-organism-response (S-O-R) model to connect SMMAs (as stimuli), brand equity (as organism), and BRL and WPP (as responses). This study was the first to use the S-O-R model to explore the effects of SMMAs on BRL and WPP in this sector of portable tech gadgets. The study's findings can guide portable tech gadget brands in Malaysia in redesigning and developing the most efficient strategies of SMMAs, which should be tailored to maximise revenues, even during any crisis period (such as the COVID-19 pandemic) when physical marketing activities are deemed difficult.

## 1 Introduction

Social media marketing is one of the fastest-growing marketing channels, offering incomparable potentials for building a brand (Zarei et al., 2021). As it is becoming more convenient and essential for business turnover, the marketing policies of almost all industries shift towards social media marketing for external advertising, promotional activities, customer relationship management, and internal employee interactions (Seo and Park, 2018). Brand managers have a vast array of opportunities to publish and share information about their brands through social media in the forms of messages, images, videos, and statements (Aljumah et al., 2021). Social media marketing can be utilised as part of a broader marketing strategy, as a consistent channel for marketing and communication, or as a series of micro-promotional events focused solely on enhancing the digital prominence of a brand (Ashley and Tuten, 2015).

Due to “social distance” and “work from home” during the COVID-19 pandemic, both developed and emerging economies saw significant drop in business activities, including physical shopping, and a rapid increase in e-commerce activities (OECD, 2020). According to a survey conducted in nine countries by the United Nations Conference on Trade and Development (UNCTAD, 2021), 52% of the surveyed consumers reported moving to online shopping since the beginning of the COVID-19 outbreak. A forecasting analysis published by McKinsey reported that the after-consequence of the COVID-19 pandemic would result in at least 60% of consumers worldwide avoiding physical shopping activities due to the crowd and the other 36% of consumers considering all new online platforms (Balloch et al., 2021). The growing number of active social media users (3.80 billion worldwide, as of January 2020) has accelerated the trend of using SMMAs to communicate with all brand stakeholders (Anggraini and Hananto, 2020). According to a recent report published by the Department of Statistics Malaysia (DOSM), participation in social networks (98% of Internet users) was identified as the most common online activity in 2020 in Malaysia, followed by searching for information about products and services (85.4%) (DOSM, 2020a). Furthermore, 93% of social media users in a prior study believed that all brands should include their presence on social media, suggesting a wide acceptance of social media marketing (Yadav and Rahman, 2018). As social networking and other online promotional tools have started to absorb a significant portion of brand marketing budgets, there are plenty of scopes for marketers to invest more time in recognising and revealing all unique and lucrative features of SMMAs more intensively (Langaro et al., 2018). Additionally, prior studies investigated the influence of brands in relation to SMMAs across a limited number of industries, including luxury brands (Kim and Ko, 2012), airlines (Seo and Park, 2018), and smartphones (Cheung et al., 2020). In order to obtain a more comprehensive understanding of SMMAs of these brands, it is essential to examine the effects of SMMAs in building a brand within various product and service contexts.

As information technology advances and electronic devices become more widely available, portable tech gadgets are becoming essential and frequently used in every sphere of life. According to the National Institute of Standards and Technology of the United States Department of Commerce, portable electronic devices are lightweight, electrically-powered, hand-held devices having the capability to store, record, and/or transmit data, text, images or video or audio data; that includes laptops, pagers, cellular telephones, compact disc radios, cassette players or recorders, audio devices, portable digital assistant, reminder recorders, and smartwatch with input capability (NIST, 2015). According to a report by Gartner, global end-user expenditure on wearable devices (i.e. smartwatch, smart-wristband, ear-worn device, head-mounted display, smart-clothing, smart-patches) increased by 18.1% from USD 69 billion in 2020 to USD 81.5 billion in 2021 (Gartner Inc., 2021). In Malaysia, the proportion of smartphone users alone increased by 0.3% from 97.9% in 2019 to 98.2% in 2020 (DOSM, 2020a). Despite the growing market trend of portable tech gadgets, there is a dearth of empirical studies exploring the crucial factors of SMMAs that can support portable tech gadget brands (in terms of brand equity and brand loyalty) to be more efficient in social media platform. Furthermore, considering the social activities and electronic commerce shifts due to the COVID-19 pandemic, this study projected that SMMAs transform and elevate the purchasing patterns of Malaysian consumers.

Organisations often adopt conventional one-way communication to build brand awareness, while social networking platforms provide two-way interaction opportunities that may facilitate shaping brand image and brand identity (Barreda et al., 2015). Furthermore, since almost 39% of social media users were reported to obtain information on various products and services from social media platforms and considering that social media is constantly being trendy by updating the latest information (Yadav and Rahman, 2018), it is critical to explore the components of SMMAs in a wide variety of industry contexts. Consumers actively participate in the purchase decision-making of their communities by expressing opinions and feedback regarding products or services on social networking sites to draw the attention of friends, encouraging acquaintances, or potential consumers (Kudeshia & Kumar, 2017). The electronic word-of-mouth distribution channels enable brands to leverage their brand image and brand awareness. One of the primary reasons consumers use social media is to acquire tailored content based on their interests and preferences, and brands should be able to provide customised information and services (Yadav and Rahman, 2018). Earlier studies established the relationships of attitudinal brand loyalty and the outputs of SMMAs, including brand advocacy (word-of-mouth), a sense of belonging in a community (interactivity), and willingness to pay premium price (Pourazad et al., 2020). Indeed, willingness to pay premium price may be a more appropriate indicator of brand success than actual purchase behaviour, as the time interval between intention to purchase and actual purchase allows other external variables to influence purchase behaviour (Augusto and Torres, 2018). Moreover, it is also important to identify consumers’ intention to pay premium price for any product during the global economic recession due to the COVID-19 pandemic.

Addressing the identified gaps in existing literature and considering the influence of SMMAs on consumers’ purchasing behaviour during the COVID-19 pandemic, this study aimed to empirically evaluate SMMAs in the context of portable tech gadget brands among Malaysian youths. The specific objectives of this study are presented as follows:

1) To identify the crucial components of SMMAs that influence the building of brand equity in terms of brand awareness and brand image
2) To examine the influence of brand equity on brand loyalty and willingness to pay premium price
3) To examine the mediating effects of brand equity on the relationships of the components of SMMAs and brand loyalty with the willingness to pay premium price

## 2 Literature review

### 2.1 Social media marketing activities (SMMAs)

Li et al. (2021a) broadly described SMMAs as organisations' advanced digital marketing operations that integrate social media networks and all parties' interactions into useful strategic ways of attaining targeted marketing productivity. In two individual studies, Mason et al. (2021a,b) examined the changes in Indian and American consumers’ purchase decision-making trends since the beginning of the COVID-19 crisis, and observed a significant rise in the widespread use of social media marketing platforms to select product requirements and for online purchasing. Kim and Ko (2012) identified entertainment, customisation, trendiness, interactivity, and word-of-mouth as factors of SMMAs, while Seo and Park (2018) applied trendiness, customisation, interaction, entertainment, and perceived risk as components of SMMAs. The current study used customisation, entertainment, trendiness, interactivity, and electronic word-of-mouth as predictors of SMMAs and explored the implications of these factors on brand loyalty, brand equity, and willingness to pay premium price among Malaysian consumers of portable tech gadgets, particularly Malaysian youths.

### 2.2 Theoretical background

**Figure 1:**
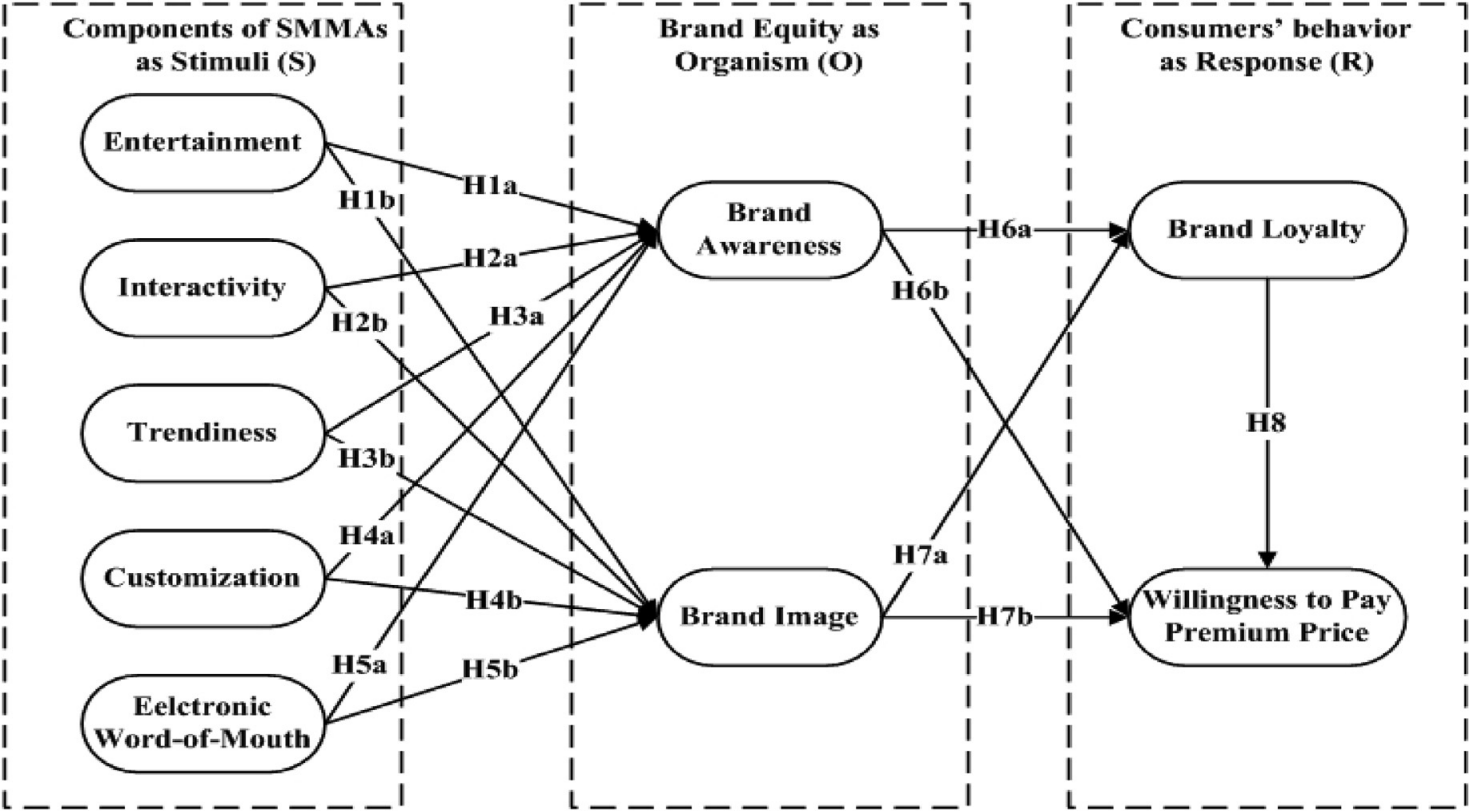
Research framework based on S-O-R theory.

Studies have explored consumers’ reactions to online marketing strategies using the stimulus-organism-response (S-O-R) model (see Zhang et al., 2014; Yadav and Rahman, 2018; Cheung et al., 2021). In support to the earlier studies, the current study employed the S-O-R model to provide a theoretical foundation for the integrated framework in Figure 1. Through this model, this study aimed to discover which components of SMMAs trigger favourable customer relationship and behavioural outcomes by influencing their cognitive and emotional responses.

Mehrabian and Russell (1974) developed the S-O-R model, which was later reformed by Jacoby (2002), stating that environmental and brand-related stimuli can influence consumers' cognitive and behavioural emotions, which in turn determine their actual behaviours. “Stimulus” includes products' features, marketing strategies, salesperson attention, and business atmosphere. “Organism”, on the other hand, refers to the consumers’ emotional and cognitive states after experiencing those stimuli. Meanwhile, “response” includes criteria like trust, commitment, purchase intention, and loyalty, as a result of those organisms (Jacoby, 2002).

In this study, aspects of SMMAs that encourage information acquisition, including customisation, entertainment, interactivity, trendiness, and marketing experiences, were examined as “stimuli” (Yadav and Rahman, 2018; Cheung et al., 2021). While a few researchers have identified EWOM as a consumer response (Seo and Park, 2018), the majority of researchers have defined it as a stimuli for brand equity and investigated it as an active component of SMMAs (Kim and Ko, 2012; Godey et al., 2016; Yadav and Rahman, 2018; Zollo et al., 2020) since consumers’ reviews through EWOM generate confidence among potential consumers to make an appropriate decision when choosing a brand. Furthermore, WOM has been defined as both stimuli and response in the same study by a few researchers (Roy et al., 2020). A majority of the researchers have recognized that consumers appear to be more motivated by EWOM such as social media reviews and recommendations, which help in making decisions regarding brands (Cheung and Thadani, 2012; Williams et al., 2019) and thus build brand awareness and brand image. Hence, the current study measured EWOM as stimuli because it better fits the tech-gadget context as well as is consistent with the majority of previous studies.

According to Keller (1993), brand equity is a mix of brand awareness and brand image. Furthermore, Lemon et al. (2001) stated that brand awareness, brand image, and corporate ethics are the core actionable dimensions of brand equity. The first step in developing brand equity is to enhance brand awareness among customers (Keller, 1993). Several scholars investigated brand equity in the context of SMMA by combining brand image and brand awareness (Godey et al., 2016; Seo and Park, 2018; Aji et al., 2020). Brand awareness and brand image were observed as “organisms” that represented consumers' emotional states after experiencing all stimuli (Yadav and Rahman, 2018; Cheung et al., 2021). In line with the earlier research, the current study operationalized "brand image" and "brand awareness" as indicators of brand equity under the organism of the S-O-R model. Finally, in order to determine "response", this study analyzed consumers’ brand loyalty and willingness to pay a premium price as a result of strong brand equity.

### 2.3 Entertainment (ENT)

ENT refers to how amusing and exciting consumers find social media marketing during shopping (Godey et al., 2016). Consumers may enjoy SMMAs more by watching videos of the brands, participating in games and contests, and getting freebies—these activities can build brand intimacy (Ashley and Tuten, 2015; France et al., 2016). Consumers use brand-related social media content for variety of entertainment, including diverting their mind from daily routine, relieving stress and emotional relief, indulging in cultural or artistic pleasure, or merely to pass time (Muntinga et al., 2011). Consumers' association with the entertaining content of a particular brand may boost their purchase intention (Dessart et al., 2015). Seo and Park (2018) recognised ENT as a vital aspect for a brand's SMMAs in entertaining consumers and building brand equity. Focusing on investigating the effects of SMMAs during the COVID-19 pandemic among Indian consumers, Mason et al. (2021b) concluded that frequently providing delightful and creative brand contents that promote pleasure and amusement results in high brand value. Based on the findings of prior studies, this study formed the following hypotheses for testing:

H1a *Entertainment, as part of SMMAs, positively influences brand awareness.*

H1b *Entertainment, as part of SMMAs, positively influences brand image.*

### 2.4 Interactivity (INT)

INT denotes the extent to which SMMAs provide multiple ways, specifically brand to consumers, consumers to brand, and consumers to consumers, of exchanging opinions and information (Dessart et al., 2015). Consumers contribute to a brand's social media by interacting and sharing ideas with other like-minded consumers to discuss the advantages and disadvantages of various products and services (Muntinga et al., 2011). Consumers and brands interact via SMMAs, regardless of time, location, or medium, resulting in friendly attention and enthusiasm for the brands (Kim and Ko, 2012). The strong engagement of consumers with a brand's SMMAs contributes to the formation of a strong brand image (France et al., 2016). Furthermore, a brand's SMMAs provide consumers the opportunities to have productive discussion and to share innovative ideas (Godey et al., 2016). By drawing customers' attention, sharing brand-related information increases consumer-brand interactions and so improves consumers' ability to perceive brands as part of their brand image (Langaro et al., 2018). Focusing on the effects of the COVID-19 pandemic on marketing strategies, Aljumah et al. (2021) reported that interactive marketing via social networking platforms contributed profound influence on the brand equity of university. Based on the findings of prior studies, this study hypothesised the following:

H2a *Interactivity, as part of SMMAs, positively influences brand awareness.*

H2b *Interactivity, as part of SMMAs, positively influences brand image.*

### 2.5 Trendiness (TRE)

TRE means providing the most up-to-date information about goods or services on social media (Godey et al., 2016). Anggraini and Hananto (2020) suggested that brands should produce the most recent content on social media to attract consumers. According to Yadav and Rahman (2018), social media users want fashion brands to provide items that are in line with modern social trends and styles. Seo and Park (2018) revealed the considerable influence of TRE on brand image and brand awareness. Through social media content, consumers analyse what products are currently being used by other consumers and obtain information to facilitate their preferences of products according to the recent trends (Yadav and Rahman, 2018). Constantly updating the latest brand materials in social media positively influences consumers’ unconscious minds by creating the sense of freshness and trendiness, contributing to brand loyalty (Zarei et al., 2021). Based on the findings of prior studies, the following hypotheses were tested in this study:

H3a *Trendiness, as part of SMMAs, positively influences brand awareness.*

H3b *Trendiness, as part of SMMAs, positively influences brand image.*

### 2.6 Customisation (CUS)

CUS discusses how well a product or service meets the needs and preferences of consumers (Seo and Park, 2018). Social media marketing customisation indicates the degree of a brand's SMMAs providing individually tailored information search options and services (Godey et al., 2016). As a result, marketers use SMMAs to convey information about consumers' favourite items, including pricing, product qualities, and features, which build brand value and trust (Cheung et al., 2020). Unlike conventional advertising, customised services rely on direct consumer engagement, fulfilling their specific requirements (Seo and Park, 2018). Customised SMMAs can influence consumers' formation of cognitive experience and brand affection, resulting in their primary preference for a particular brand when they make a purchasing decision (Dessart et al., 2015; Cheung et al., 2020). Seo and Park (2018) found CUS as the second-most influential component of SMMAs that affects brand equity. Based on the findings of prior studies, the following hypotheses were proposed for testing:

H4a *Customisation, as part of SMMAs, positively influences brand awareness.*

H4b *Customisation, as part of SMMAs, positively influences brand image.*

### 2.7 Electronic word-of-mouth (EWOM)

The extent of EWOM is related to how consumers share and publish content regarding brand experience through social media (Kudeshia & Kumar, 2017). Consumers are heavily influenced and guided by online reviews and/or ratings, which are a key source of WOM (Cheung and Thadani, 2012). When negative exogenous EWOM is spread on social media, it can be extremely damaging to a brand's reputation (Williams et al., 2019). Li et al. (2021a) recognised the effectiveness of the positive actions of a brand, such as publicly apologise, implement problem-solving approaches, and provide flexible support, in minimising negative EWOM. The influence of EWOM on brand equity is significantly stronger than conventional word-of-mouth due to its ease of use, timeliness, place independence, and easy accessibility (Farzin et al., 2021). Consumers rely on EWOM biases for product and service selection since EWOM is kept in reference to prospective customers throughout the whole purchase process (Roy et al., 2020). Meanwhile, Mason et al. (2021b) observed the sharp rise of the use of social media to publish reviews regarding purchase experiences (such as disappointment, happiness, or satisfaction) since the beginning of the COVID-19 pandemic among Indian consumers. Thus, the current study postulated the following hypothesis:

H5a *Electronic word-of-mouth, as part of SMMAs, positively influences brand awareness.*

H5b *Electronic word-of-mouth, as part of SMMAs, positively influences brand image.*

### 2.8 Brand awareness (BBA)

BBA is an approach for consumers to become aware of, acquainted with, and remember a particular brand (Barreda et al., 2015). SMMAs may help raise BBA and establish favourable brand image by allowing brands to connect with prospective and existing consumers (Seo and Park, 2018). As time and place are not constraints for SMMAs, the presence of a particular brand on social media platforms may effectively educate, familiarise, and elevate brand awareness (O'Flynn, 2017). According to Barreda et al. (2015), strong BBA affects other brand-related factors, including brand image and brand loyalty. Moreover, Anselmsson et al. (2014) found the positive influence of BBA on brand loyalty and willingness to pay premium price. Consumers who recognise a brand's logo are more likely to suggest the brand and ready to pay premium price for its products or services (Hyun and Kim, 2011). Prior studies also revealed that a high level of brand awareness may help a brand to generate premium price in the marketplace (Bougenvile and Ruswanti, 2017). The findings of prior studies led to the formation of the following hypotheses in the current study:

H6a *Brand awareness positively influences brand loyalty.*

H6b *Brand awareness positively influences willingness to pay premium price.*

### 2.9 Brand image (BBI)

BBI refers to consumers' views about a brand (Keller, 1993). A positive brand image should have an extra advantage and beneficial impact on customer behaviour, whereas a negative brand image would indirectly promote negative consumer attitudes, resulting in the majority of consumers refusing to engage with that brand (Kazmi and Mehmood, 2016). Li et al. (2021b) postulated the influence of a strong BBI on consumers' perceptions, resulting in higher brand loyalty. A favorable brand image should be readily accepted by consumers, resulting in enhanced customer satisfaction and brand loyalty (Savitri et al., 2022). A company with a strong brand image would be able to rapidly and successfully implement promotional strategies, strengthening consumer loyalty, whilst a company with a poor brand image would indeed do the opposite (Dash et al., 2021). Furthermore, Yodpram and Intalar (2020) suggested the direct influence of brand image on consumers’ willingness to pay premium price. Consumers who have a favourable perception of a brand are prepared to pay a premium price to attain the brand (Keller, 1993). Earlier studies empirically exhibited that vastly communicated corporate brand identity contributes to broadening brand image and reputation, resulting in higher willingness to pay premium price among consumers (Farzin et al., 2021). Based on the above findings, the current study derived the following hypotheses:

H7a *Brand image positively influences brand loyalty.*

H7b *Brand image positively influences willingness to pay premium price.*

### 2.10 Brand loyalty (BRL)

BRL refers to consumers repurchase intention and commitment to purchase a specific brand over other brands (Nyadzayo and Khajehzadeh, 2016). Prior studies on luxury brands identified BRL as the degree to which consumers express their intention to purchase the brand or their actual purchase of the brand (Godey et al., 2016). Laroche et al. (2013) argued that SMMAs can improve consumer relationships and in turn develop BRL. Yodpram and Intalar (2020) postulated the direct relationships of BRL and BBI with consumers’ willingness to pay premium price. In a prior study, Pourazad et al. (2020) empirically demonstrated the positive influence of brand loyalty on willingness to pay premium price. In line with the findings of prior studies, this study proposed the following hypothesis:

H8 *Brand loyalty positively influences willingness to pay premium price.*

### 2.11 Willingness to pay premium price (WPP)

A brand gains premium pricing opportunity when the total number of consumers who are ready to pay higher for a product exceeds the total number of consumers who are willing to pay for a similar product from other brands (Porral et al., 2013). Interactions in social media platforms may influence consumers’ readiness to pay premium price for a particular brand (Augusto and Torres, 2018). Earlier studies suggested that consumers are ready to pay premium for non-functional advantages, such as psychological and sensory satisfaction, which allow them to have emotional and hedonic experiences from a particular brand (Astakhova et al., 2017; Pourazad et al., 2020). Augusto and Torres (2018) demonstrated that WPP signifies the strength of a brand in the industry. Numerous empirical studies established the direct influence of higher brand equity on the capacity of the brand to claim higher pricing than its competitors (Anselmsson et al., 2014; Bougenvile and Ruswanti, 2017; Farzin et al., 2021; Zarei et al., 2021). Therefore, this study postulated the positive effects of BBA, BBI, and BRL via SMMAs on WPP among Malaysian consumers of portable tech gadgets.

All associations hypothesized and examined in this study, presented in Figure 1 below:

## 3 Methodology

### 3.1 Population, sample size, and data collection

Using the cross-sectional research design, the current study quantitatively examined the effects of SMMAs on brand equity and WPP for portable tech gadgets among Malaysian youths. According to the International Labour Organisation (ILO, 2013), youths and working adults are classified as individuals of between the ages of 18 and 40. Based on the most recent official census performed in 2020, 12.1 million Malaysian youths aged 15 to 40 accounted for 37.12% of the country's overall population (DOSM, 2020b). Based on this sampling frame, using G-Power (version 3.1.9), the calculated sample size was 160, with power of 0.95 and effect size of 0.15 for eight predictors (Faul et al., 2007). Nonetheless, this study gathered data from 1,322 Malaysian youths in order to mitigate any potential complications stemming from a small sample size. The survey was conducted online via Google Forms. The link to the online questionnaire was disseminated to all respondents over social media platforms (Facebook, Instagram, LinkedIn, WhatsApp), as well as via email. Complete data are available as supplemental materials with this article (DATA - SMMA and Portable Tech Gadgets.csv).

Informed consent for participation was obtained from respondents who participated in the survey. For the respondents who participated the survey online (using google form), they were asked to read the ethical statement posted on the top of the form (*There is no compensation for responding nor is there any known risk. In order to ensure that all information will remain confidential, please do not include your name. Participation is strictly voluntary and you may refuse to participate at any time*) and proceed only if they agree. No data was collected from anyone under 18 years old.

### 3.2 Instrument

With minimal modifications, the current study adapted previously tested and validated instruments. The questionnaire was designed using simple and unbiased wordings to allow the respondents to understand all questions easily. Items that measured ENT in this study were adopted from studies by Kim and Ko (2010), Godey et al. (2016), and Seo and Park (2018). INT, TRE, CUS, and EWOM were measured using five items, respectively. All of these items were taken from studies by Godey et al. (2016), Seo and Park (2018), and Yadav and Rahman (2018). Items that gauged BBA and BBI were adopted from studies by Godey et al. (2016) and Seo and Park (2018). Next, five items that measured BRL in this study were derived from studies by Laroche et al. (2013) and Godey et al. (2016). Finally, four items that measured WPP were obtained from studies by Godey et al. (2016) and Yazdanparast et al. (2016). All items, except the last item of WPP, were measured using a seven-point Likert scale, with the endpoints of “strongly disagree” (1) and “strongly agree” (7). The last item of WPP was measured using the following five options: 0%, 5%, 10%, 15%, and 20% and more. Complete questionnaire presented in Appendix 1.

### 3.3 Common method variance (CMV)

In this study, the single factor accounted for 42.539% (below the recommended threshold of 50%), which approved the inconsequential influence of CMV (Podsakoff et al., 2012). Additionally, CMV evaluated the current study by testing the full collinearity for all constructs (Kock, 2015). All constructs regressed on the common variable. The recorded values of variance inflation factor (VIF) for BBA (3.383), BBI (3.596), BRL (3.284), CUS (3.537), EWOM (2.918), ENT (2.430), INT (2.810), TRE (3.296), and WPP (1.955) were less than 3.3, suggesting the absence of bias from the single-source data (Kock, 2015).

### 3.4 Data analysis methods

Partial least squares structural equation modelling (PLS-SEM) is a causal modelling technique that optimises the explained variance of endogenous latent components (Hair et al., 2017). Variance-based PLS-SEM estimation was performed in this study because the nature of this study was exploratory with non-normality issues. As per the recommendation by Hair et al. (2019), the current study presented the results of (1) descriptive analysis (mean and standard deviation), (2) internal reliability consistency (Cronbach's alpha, Dijkstra-Hensele's rho, and composite reliability), (3) convergent validity (average variance extracted (AVE)), (4) discriminant validity (Fornell-Larcker criterion, loadings, and cross-loadings), (5) coefficient of determination (*r*^*2*^), (6) effect size (*f*^*2*^), (7) path coefficient (*β*), and (8) predictive relevance (*Q*^*2*^).

## 4 Results

### 4.1 Demographic characteristics of respondents

Table 1 presents the demographic profile of respondents in this study. Out of 1,332 respondents, the majority of the respondents (52.1%) were female. About 50.5% of the total respondents were between the ages of 21 and 25, followed by those of the ages of below 21 (39.8%). Besides that, most of the respondents (79.3%) reported monthly income of below RM 2,500, followed by those with monthly income of between RM 2,501 and RM 5,000 (14.1%). In addition, 93.4% of the total respondents were single, and the other 5.5% of total respondents were married. Furthermore, majority of respondents (60.5%) indicated to have Bachelor's degree or equivalent degree, followed by secondary school certificate (19.1%) and diploma or technical-level education (17.4%). Finally, 86.9% of the total respondents reported residing in urban areas, while the remaining respondents indicated residing in rural areas.

**Table 1:** Demographic characteristics.

	N	%		N	%
*Gender*			*Marital Status*		
Male	633	47.9	Single	1235	93.4
Female	689	52.1	Married	73	5.5
Total	1322	100.0	Divorced	10	.8
			Widowed	4	.3
*Age Group*			Total	1322	100.0
Below 21 years	526	39.8			
21–25 years	667	50.5	*Education*		
26–30 years	60	4.5	Secondary school certificate	253	19.1
31–35 years	32	2.4	Diploma/technical school certificate	230	17.4
36–40 years	37	2.8	Bachelor degree or equivalent	800	60.5
Total	1322	100.0	Master's degree	37	2.8
			Doctoral degree	2	.2
*Average Monthly Income* (RM)			Total	1322	100.0
Below RM2500	1048	79.3			
RM2501-RM5000	187	14.1	*Living Area*		
RM5001-RM7500	43	3.3	Urban	1149	86.9
RM7501-RM10,000	17	1.3	Rural	173	13.1
RM10,001-RM12500	5	.4	Total	1322	100.0
More than RM12500	22	1.7			
Total	1322	100.0			

### 4.2 Validity and reliability

As part of the reliability analysis, Cronbach's alpha, reflecting the inter-correlations of indicators, should exceed the threshold value of more than 0.7 (Hair et al., 2017). As shown in Table 2, all indicators in this study recorded Cronbach's alpha values of more than 0.720, indicating their reliability. Besides that, Dijkstra-Hensele's rho results revealed that all values surpassed the threshold value of 0.7, which substantially supported the high reliability of all constructs. Furthermore, CR has been widely employed as an alternate measure of internal consistency, with a cut-off value greater than 0.7 (Hair et al., 2019). As shown in Table 2, the recorded values of CR for all items exceeded 0.825, which confirmed the high level of reliability. AVE is commonly used to evaluate convergent validity, which is a measure of how much of the variance in indicators can be explained by the latent variable. As recommended by Hair et al. (2017), the recorded values of AVE for all constructs exceeded 0.563 (as presented in Table 2), which indicated high convergent validity.

**Table 2:** Validity and Reliability of components.

Variables	No. Items	Mean	Standard Deviation	Cronbach's Alpha	Dijkstra-Hensele's *rho*	Composite Reliability	Average Variance Extracted	Variance Inflation Factor
BBA	5	3.742	0.981	0.872	0.874	0.908	0.663	3.091
BBI	5	3.725	0.954	0.888	0.889	0.918	0.691	3.442
BRL	5	5.066	1.335	0.861	0.864	0.900	0.644	2.394
CUS	5	3.888	0.879	0.842	0.843	0.888	0.613	3.453
EWOM	5	3.798	0.934	0.854	0.854	0.896	0.632	2.398
ENT	5	4.089	0.810	0.857	0.863	0.898	0.639	2.388
INT	5	3.985	0.864	0.831	0.834	0.880	0.596	2.799
TRE	5	3.932	0.890	0.845	0.848	0.890	0.618	3.238
WPP	4	4.510	1.499	0.720	0.811	0.825	0.563	-

**Note:** BBA: Brand Awareness; BBI: Brand Image; BRL: Brand Loyalty; CUS: Customization; EWOM: Electronic Word-of-Mouth; ENT: Entertainment; INT: Interactivity; TRE: Trendiness; WPP: Willingness to Pay Premium Price.

**Source:** Authors’ data analysis

In order to analyse the Fornell-Larcker criterion, the correlations of latent variables were compared to the square root of the recorded AVE values in this study. The square root of each construct's AVE must be higher than the highest correlation with any other variable (Hair et al., 2017). The results in Table 3 revealed that all elements were able to match the required specifications of the Fornell-Larcker criterion. For the evaluation of discriminant validity, cross-loadings were also examined. In order to evaluate the appropriateness of the model, the outer loading of an indicator for the related construct should exceed any of its cross-loadings (correlation) with other constructs (Hair et al., 2017). The values of loadings and cross-loadings in Table 4 demonstrated that all items had maximum loading with their respective constructs, which satisfied the necessary requirements.

**Table 3:** Fornell-Larcker criterion.

	BBA	BBI	BRL	CUS	EWOM	ENT	INT	TRE	WPP
BBA	0.814								
BBI	0.807	0.831							
BRL	0.705	0.741	0.803						
CUS	0.623	0.631	0.624	0.783					
EWOM	0.685	0.667	0.677	0.730	0.795				
ENT	0.545	0.518	0.555	0.668	0.613	0.799			
INT	0.557	0.546	0.549	0.727	0.638	0.704	0.772		
TRE	0.591	0.589	0.617	0.780	0.686	0.690	0.725	0.786	
WPP	0.567	0.620	0.741	0.508	0.566	0.450	0.455	0.499	0.751

Note: BBA: Brand Awareness; BBI: Brand Image; BRL: Brand Loyalty; CUS: Customization; EWOM: Electronic Word-of-Mouth; ENT: Entertainment; INT: Interactivity; TRE: Trendiness; WPP: Willingness to Pay Premium Price.

Source**:** Authors’ data analysis

**Table 4:** Loadings and cross-loadings.

Code	BBA	BBI	BRL	CUS	ENT	INT	TRE	EWOM	WPP
BBA1	**0.771**	0.624	0.525	0.492	0.439	0.442	0.456	0.532	0.435
BBA2	**0.807**	0.642	0.578	0.550	0.484	0.491	0.511	0.587	0.453
BBA3	**0.812**	0.647	0.578	0.475	0.399	0.437	0.460	0.545	0.458
BBA4	**0.844**	0.682	0.595	0.516	0.454	0.449	0.495	0.574	0.479
BBA5	**0.835**	0.687	0.593	0.504	0.443	0.449	0.482	0.549	0.484
BBI1	0.746	**0.809**	0.601	0.533	0.416	0.447	0.495	0.567	0.511
BBI2	0.658	**0.825**	0.590	0.485	0.405	0.429	0.443	0.505	0.488
BBI3	0.664	**0.851**	0.619	0.503	0.408	0.440	0.474	0.553	0.507
BBI4	0.662	**0.844**	0.623	0.556	0.455	0.484	0.509	0.569	0.526
BBI5	0.625	**0.827**	0.643	0.542	0.463	0.467	0.519	0.574	0.541
BRL1	0.623	0.695	**0.801**	0.533	0.481	0.465	0.531	0.577	0.565
BRL2	0.596	0.605	**0.848**	0.533	0.458	0.459	0.506	0.577	0.595
BRL3	0.578	0.582	**0.811**	0.510	0.461	0.436	0.491	0.566	0.580
BRL4	0.551	0.584	**0.799**	0.466	0.419	0.429	0.452	0.523	0.653
BRL5	0.473	0.494	**0.752**	0.457	0.403	0.412	0.495	0.465	0.581
CUS1	0.477	0.454	0.457	**0.778**	0.526	0.564	0.620	0.542	0.360
CUS2	0.490	0.482	0.487	**0.787**	0.535	0.574	0.616	0.545	0.383
CUS3	0.498	0.503	0.483	**0.786**	0.506	0.550	0.596	0.560	0.416
CUS4	0.492	0.522	0.513	**0.796**	0.532	0.567	0.638	0.616	0.422
CUS5	0.482	0.505	0.499	**0.767**	0.517	0.592	0.583	0.590	0.404
ENT1	0.452	0.415	0.463	0.529	**0.828**	0.554	0.562	0.489	0.382
ENT2	0.443	0.418	0.451	0.560	**0.838**	0.583	0.576	0.494	0.374
ENT3	0.445	0.420	0.450	0.533	**0.815**	0.572	0.551	0.479	0.363
ENT4	0.467	0.457	0.479	0.571	**0.815**	0.577	0.582	0.557	0.389
ENT5	0.365	0.349	0.364	0.470	**0.692**	0.530	0.482	0.419	0.278
INT1	0.380	0.365	0.410	0.541	0.585	**0.733**	0.515	0.435	0.310
INT2	0.394	0.389	0.406	0.543	0.553	**0.784**	0.556	0.482	0.339
INT3	0.437	0.441	0.422	0.553	0.523	**0.790**	0.551	0.467	0.361
INT4	0.462	0.451	0.432	0.576	0.535	**0.755**	0.578	0.535	0.364
INT5	0.465	0.450	0.445	0.590	0.531	**0.796**	0.591	0.533	0.373
TRE1	0.435	0.434	0.453	0.613	0.517	0.563	**0.754**	0.503	0.354
TRE2	0.471	0.457	0.490	0.586	0.536	0.558	**0.790**	0.525	0.420
TRE3	0.456	0.454	0.500	0.599	0.543	0.554	**0.798**	0.535	0.390
TRE4	0.505	0.516	0.504	0.617	0.568	0.588	**0.812**	0.586	0.427
TRE5	0.451	0.446	0.473	0.652	0.548	0.587	**0.776**	0.544	0.366
EWOM1	0.524	0.521	0.544	0.627	0.512	0.525	0.588	**0.785**	0.460
EWOM2	0.530	0.540	0.558	0.635	0.532	0.547	0.590	**0.807**	0.464
EWOM3	0.535	0.519	0.536	0.553	0.453	0.483	0.513	**0.807**	0.432
EWOM4	0.555	0.533	0.539	0.577	0.494	0.528	0.537	**0.808**	0.438
EWOM5	0.576	0.536	0.512	0.511	0.444	0.454	0.501	**0.768**	0.453
WPP1	0.487	0.513	0.621	0.453	0.392	0.400	0.446	0.485	**0.766**
WPP2	0.476	0.515	0.622	0.402	0.381	0.372	0.405	0.467	**0.877**
WPP3	0.491	0.551	0.653	0.447	0.378	0.394	0.427	0.497	**0.883**
WPP4	0.128	0.165	0.173	0.112	0.107	0.097	0.118	0.122	**0.344**

**Note:** BBA: Brand Awareness; BBI: Brand Image; BRL: Brand Loyalty; CUS: Customization; EWOM: Electronic Word-of-Mouth; ENT: Entertainment; INT: Interactivity; TRE: Trendiness; WPP: Willingness to Pay Premium Price.

**Source:** Authors’ data analysis

### 4.3 Path analysis

Table 5 presents the results of the testing of hypotheses on the relationships of all components of SMMAs with BBA. The results revealed statistically significant and positive effects of ENT (*β* = 0.090, *p* = 0.009), TRE (*β* = 0.077, *p* = 0.047), CUS (*β* = 0.146, *p* = 0.001), and EWOM (*β* = 0.437, *p* = 0.000) on BBA. Therefore, H1a, H3a, H4a, and H5a were supported. INT recorded positive *β*-value of 0.053, but statistically insignificant p-value of 0.111. This indicated that INT had no substantial influence on BBA in this study. Thus, H2a was rejected.

**Table 5:** Path analysis.

Hypo		Beta	CI-Min	CI-Max	*t*	*p*	*r*^2^	*f*^*2*^	*Q*^*2*^	Decision
H1a	ENT→BBA	0.090	0.032	0.151	2.372	0.009	0.513	0.001	0.508	Supported
H2a	INT→BBA	0.053	-0.033	0.111	1.221	0.111		0.002		Rejected
H3a	TRE→BBA	0.077	-0.001	0.150	1.676	0.047		0.004		Supported
H4a	CUS→BBA	0.146	0.056	0.215	3.036	0.001		0.013		Supported
H5a	EWOM→BBA	0.437	0.377	0.492	12.029	0.000		0.164		Supported
H1b	ENT→BBI	0.037	-0.025	0.095	1.035	0.151	0.495	0.001	0.489	Rejected
H2b	INT→BBI	0.047	-0.036	0.109	1.066	0.144		0.002		Rejected
H3b	TRE→BBI	0.093	0.023	0.170	2.042	0.021		0.005		Supported
H4b	CUS→BBI	0.210	0.137	0.287	4.596	0.000		0.025		Supported
H5b	EWOM→BBI	0.398	0.335	0.466	9.782	0.000		0.131		Supported
H6a	BBA→BRL	0.309	0.249	0.369	8.358	0.000	0.582	0.080	0.486	Supported
H7a	BBI→BRL	0.492	0.432	0.549	13.675	0.000		0.202		Supported
H6b	BBA→WPP	0.193	-0.063	0.057	4.815	0.000	0.559	0.000	0.331	Supported
H7b	BBI→WPP	0.464	0.086	0.224	11.312	0.000		0.016		Supported

H8	BRL→WPP	0.625	0.575	0.678	20.564	0.000		0.371		Supported

**Note:** BBA: Brand Awareness; BBI: Brand Image; BRL: Brand Loyalty; CUS: Customization; EWOM: Electronic Word-of-Mouth; ENT: Entertainment; INT: Interactivity; TRE: Trendiness; WPP: Willingness to Pay Premium Price.

**Source:** Authors’ data analysis

The obtained results also revealed statistically significant and positive effects of TRE (*β* = 0.093, *p* = 0.021), CUS (*β* = 0.210, *p* = 0.000), and EWOM (*β* = 0.398, *p* = 0.000) on BBI. Thus, H3b, H4b, and H5b were supported. ENT (*β* = 0.037, *p* = 0.151) and INT (*β* = 0.047, *p* = 0.144) were found to have no significant impact on BBI. Therefore, H1b and H2b were rejected.

Table 5 also presents the results on the relationships of BBA and BBI with BRL. The results depicted statistically significant and positive effects of BBA (*β* = 0.303, *p* = 0.000) and BBI (*β* = 0.492, *p* = 0.000) on BRL. Therefore, the study supported both H6a and H7a.

Finally, the results on the relationships of BBA, BBI, and BRL with WWP are presented in Table 5. All BBA, BBI, and BRL recorded positive *β*-values (0.193, 0.464, and 0.625, respectively) and significant *p*-values (of 0.000). The results confirmed statistically significant and positive effects of BBA, BBI, and BRL on WPP for portable tech gadgets among Malaysian youths. Thus, H6b, H7b, and H8 were also supported.

Moreover, in order to measure the predictive capacity of the model, coefficient of determination (*r*^*2*^), which reflects the amount of variation in endogenous constructs explained by all related exogenous constructs (Hair et al., 2017), was considered in this study. Endogenous latent variables with *r*^*2*^ of 0.75, 0.50, or 0.25 are classified as significant, medium, or weak, respectively (Hair et al., 2017). The recorded *r*^*2*^ value (0.513) for BBA implied that the components of SMMAs explained a considerable proportion (51.3%) of the variance in Malaysian consumers brand awareness towards portable tech devices. Similarly, the components of SMMAs explained moderate proportion (49.5%) of the variance in BBI. About 58.2% of the variance in BRL was explained through BBA and BBI. Finally, BBA, BBI, and BRL were found to explain moderate portion of variance (55.9%) in WPP.

Apart from that, *Q*^*2*^ value was also considered as a criterion of predictive accuracy (Hair et al., 2017) in this study. *Q*^*2*^ of greater than zero in a structural model for a given reflective endogenous latent variable suggests the model's predictive relevance for a specific dependent variable (Hair et al., 2017). Referring to Table 5, all values of *Q*^*2*^ exceeded zero, which indicated substantial predictive relevance for all factors.

### 4.4 Mediating effects

Table 6 presents the results on the mediating effects of BBA, BBI, and BRL. At the first level, the mediating effects of BBA and BBI on the relationships of all five components of SMMAs with BRL were demonstrated. BBA was found to significantly and positively influence the relationships of ENT (*β* = 0.028, *p* = 0.014), CUS (*β* = 0.045, *p* = 0.002), and EWOM (*β* = 0.135, *p* = 0.000) with BRL. However, BBA did not mediate the relationships of INT (*β* = 0.016, *p* = 0.112) and TRE (*β* = 0.024, *p* = 0.050) with BRL. Meanwhile, BBI was revealed to significantly and positively mediate the relationships of TRE (*β* = 0.046, *p* = 0.023), CUS (*β* = 0.103, *p* = 0.000), and EWOM (*β* = 0.196, *p* = 0.000) with BRL. At the same time, BBI did not mediate the relationships of ENT (*β* = 0.018, *p* = 0.115) and INT (*β* = 0.023, *p* = 0.145) with BRL.

**Table 6:** Mediating effects.

Associations	Beta	CI -Min	CI -Max	*t*	*p*	Decision
ENT→BBA→BRL	0.028	0.010	0.050	2.215	0.014	Supported
INT→BBA→BRL	0.016	-0.009	0.035	1.216	0.112	Rejected
TRE→BBA→BRL	0.024	0.000	0.047	1.644	0.050	Rejected
CUS→BBA→BRL	0.045	0.020	0.071	2.859	0.002	Supported
EWOM→BBA→BRL	0.135	0.104	0.168	6.737	0.000	Supported
ENT→BBI→BRL	0.018	-0.010	0.046	1.031	0.151	Rejected
INT→BBI→BRL	0.023	-0.017	0.056	1.057	0.145	Rejected
TRE→BBI→BRL	0.046	0.012	0.086	2.003	0.023	Supported
CUS→BBI→BRL	0.103	0.068	0.146	4.360	0.000	Supported
EWOM→BBI→BRL	0.196	0.152	0.235	7.780	0.000	Supported
ENT→BBA→WPP	0.000	-0.005	0.005	0.010	0.496	Rejected
INT→BBA→WPP	0.000	-0.004	0.005	0.008	0.497	Rejected
TRE→BBA→WPP	0.000	-0.006	0.005	0.008	0.497	Rejected
CUS→BBA→WPP	0.000	-0.009	0.009	0.010	0.496	Rejected
EWOM→BBA→WPP	0.000	-0.027	0.024	0.010	0.496	Rejected
ENT→BBI→WPP	0.006	-0.002	0.017	0.957	0.170	Rejected
INT→BBI→WPP	0.007	-0.004	0.020	0.976	0.165	Rejected
TRE→BBI→WPP	0.015	0.004	0.033	1.664	0.048	Supported
CUS→BBI→WPP	0.033	0.018	0.056	2.841	0.002	Supported
EWOM→BBI→WPP	0.062	0.036	0.095	3.471	0.000	Supported
BBA→BRL→WPP	0.193	0.157	0.238	7.667	0.000	Supported
BBI→BRL→WPP	0.307	0.265	0.351	11.384	0.000	Supported

**Note:** BBA: Brand Awareness; BBI: Brand Image; BRL: Brand Loyalty; CUS: Customization; EWOM: Electronic Word-of-Mouth; ENT: Entertainment; INT: Interactivity; TRE: Trendiness; WPP: Willingness to Pay Premium Price.

**Source:** Authors’ data analysis

At the second level, the mediating effects of BBA and BBI on the relationships of all five components of SMMAs with WPP were examined. Referring to Table 6, BBA was found to have no substantial mediating effects on any of the relationships of SMMAs with WPP. On the other hand, BBI positively and significantly mediated the relationships of TRE (*β* = 0.015, *p* = 0.048), CUS (*β* = 0.033, *p* = 0.002), and EWOM (*β* = 0.062, *p* = 0.000) with WPP. In contrast, BBI was found to have no substantial mediating effects on the relationships of ENT (*β* = 0.006, *p* = 0.170) and INT (*β* = 0.007, *p* = 0.165) with WPP. At the third level, the mediating effects of BRL on the relationships of BBA and BBI with WPP were assessed. For both relationships, BRL was found to exhibit significant mediating effects.

### 4.5 Importance-performance factors

An importance-performance matrix analysis was also conducted on all eight constructs and WPP. The analysis aimed to identify which components of SMMAs are more influential among Malaysian youths in the context of portable tech gadget users. Table 7 shows the results of the importance-performance matrix analysis. ETT was identified as the most crucial factor with the highest performance value of 77.167, followed by INT (74.534), TRE (73.301), CUS (72.193), and EWOM (70.044). For effects, the most crucial factor was BRL, with the total effect value of 0.625, followed by BBI (0.464), EWOM (0.269), and lastly, BBA (0.193).

**Table 7:** Importance-performance matrix.

Target Construct Variables	Willingness to pay a premium price	
	Total Effect	Performance
ENT	0.034	77.167
INT	0.032	74.534
TRE	0.058	73.301
CUS	0.125	72.193
EWOM	0.269	70.044
BBA	0.193	68.554
BBI	0.464	68.137
BRL	0.625	67.745

**Note:** BBA: Brand Awareness; BBI: Brand Image; BRL: Brand Loyalty; CUS: Customization; EWOM: Electronic Word-of-Mouth; ENT: Entertainment; INT: Interaction; TRE: Trendiness; WPP: Willingness to Pay Premium Price.

**Source:** Authors’ data analysis

## 5 Discussion

Based on the obtained findings of this study, the components of SMMAs played significant roles of improving brand equity, specifically BBA and BBI. ENT, TRE, CUS, and EWOM were found to be primary drivers that enhance both BBA and BBI. Moreover, BBA and BBI contributed significant and positive effects on BRL, which largely affected WPP among Malaysian consumers of portable tech gadgets.

Furthermore, the obtained results of this study revealed the significant and positive influence of ENT on BBA, which corroborated the findings of prior studies (Godey et al., 2016; Seo and Park, 2018). As all physical shopping activities were prohibited during the COVID-19 crisis, Malaysian consumers of portable tech gadgets were unable to experience the pleasure and satisfaction of physical purchase. As a result, they were attracted to the promotional and entertaining offers from brands, as part of their SMMAs. On the other hand, ENT was found to have no significant impact on BBI, which was also revealed to be consistent with the findings reported by Cheung et al. (2020) and Zollo et al. (2020). In the context of social media, although entertaining content of brands may well be appealing, the entertainment value of reading such content is derived from consumers’ passive appreciation. As a result, the entertainment value in the search phase is considered as passive preference that is self-oriented (Cheung et al., 2020). This suggests that, for consumers of portable tech gadgets in Malaysia, passive pleasure or entertaining resources of brands on social media platforms are less attractive and effective in enhancing brand image because brand image requires practical and effort-intensive activities.

Besides that, the obtained results revealed insignificant relationships of INT with BBA and BBI, which were found to be contradictory with the results of the majority of prior studies (Yadav and Rahman, 2018; Aji et al., 2020; Zollo et al., 2020). The possible explanation for such findings lies in the lack of interest among Malaysian consumers to connect directly with portable tech gadget brands, and also vice versa. Alternatively, Malaysian consumers of portable tech gadgets may have encountered insufficiency on whom they may discuss and evaluate product aspects via social media interactions with other like-minded consumers.

The significant and positive effects of TRE, CUS, and EWOM on BBA and BBI were found to be analogous to the reported results of earlier studies (Godey et al., 2016; Seo and Park, 2018; Yadav and Rahman, 2018; Zollo et al., 2020; Aji et al., 2020). TRE should be utilised as a tool to increase consumers’ cognitive processing and attachment with a particular brand (Dessart et al., 2015; Kudeshia & Kumar, 2017). Due to the COVID-19 pandemic, physical official meetings, face-to-face purchase consultations, and physical product demonstration are limited. As a result, Malaysian consumers are completely reliant on the trendiness of brands to provide up-to-date information on their products and services through social media platforms. Furthermore, numerous brands have started SMMAs during the COVID-19 crisis, and consumers have the options and access to a substantial amount of information available on social media. Hence, consumers are attracted solely to brands that can offer customised search options and tailored product solutions. Additionally, as social physical get-togethers were banned during the COVID-19 crisis, Malaysian consumers found social media to be a more convenient tool to express and publish their feedback, product experiences, and brand views. As a result, consumers have become more dependent on EWOM via social media, and they become increasingly engaged in analysing EWOM before choosing a brand.

The results of this study also revealed the significant and positive influence of BBA and BBI on both BRL and WPP, which were supported by several previous studies (Anselmsson et al., 2014; Bougenvile and Ruswanti, 2017; Augusto and Torres, 2018; Zarei et al., 2021). These findings confirmed the influence of brand equity on Malaysian consumers to express their personality via a brand. In order to create good image, consumers are ready to pay premium price for a particular brand over the equal status of other brands. Moreover, in order to meet consumers' demand for being unique and novel, strong brand equity helps to develop the feeling of belonging to a prestigious community among consumers. The current study's results also confirmed the positive influence of strong BRL on WPP, which was consistent with the findings reported by Pourazad et al. (2020). This indicates that, even in a financial downturn circumstance, such as the COVID-19 pandemic, strong BRL can alleviate Malaysian consumers' indecisiveness and confusion for paying premium price for portable tech gadgets.

## 6 Implications of study

### 6.1 Theoretical implications

Earlier studies confirmed the importance of social media in driving community engagement (Cheung et al., 2020), consumer-brand relationships (Laroche et al., 2013), and brand equity (Godey et al., 2016; Yadav and Rahman, 2018). However, there is a scarcity of studies that investigate the effects of SMMAs on brand loyalty. Likewise, the relationship between SMMAs and willingness to pay a premium price is rarely examined, indicating another gap in the existing literature. Finally, no studies have been conducted in Malaysia to investigate the impact of SMMAs on Malaysian consumers’ responses towards portable tech-gadgets in terms of brand loyalty and willingness to pay premium prices. By bridging the preceding gaps, this study significantly contributes to the existing marketing and branding literature in the following manner: Firstly, this is the first study of its kind to consider the impacts of SMMA on portable tech-gadgets and identify the outcomes for brand loyalty and willingness to pay premium prices. Secondly, the current study is one of the pioneers to use the S–O–R model to directly link individual components of the SMMAs, giving this research a robust theoretical base.

More specifically, the current study used S-O-R theory to directly connect antecedents of SMMAs, conceptualizing those as consumers' inner states of stimuli, which drive consumers’ responses to brand loyalty and willingness to pay premium prices. Furthermore, SMMA has not been thoroughly studied during a catastrophic circumstance such as COVID-19, while the current study bridged this research gap by collecting, analysing, and interpreting results considering the COVID-19 lockdown context to evaluate the effectiveness of the theory in an unfriendly environment. Most importantly, the current study framework would be useful for evaluating SMMA components' effectiveness for enhancing branding operations and eventually inducing consumers' willingness to pay premium prices for other smart gadget sectors such as IoT devices, health and fitness trackers, wearable payment devices, and so on.

### 6.2 Managerial implications

The findings of this study presented practical and managerial implications by revealing the real views of SMMAs and identifying the most important components of SMMAs that influence Malaysian consumers' BRL and WPP for portable tech gadgets. Using the findings of this research, portable tech gadget brands may build the most effective marketing strategies that are specifically tailored for the Malaysian market. Moreover, the findings can help other tech-device firms that intend to switch from conventional marketing to SMMAs. This study concluded that firms should actively encourage consumers to use social media by introducing more attractive add-ons and premium services in social media. Consumers may experience discomfort as a result of poor or negative experiences with brands' SMMAs that may lead them to switch brands. This study found that ENT, as part of SMMAs, can be a crucial factor for increasing brand awareness, implying that marketers should concentrate on deploying materials that can capture consumers' hedonic perception. Malaysian consumers of portable tech-gadgets are more responsive to this component when compared to other smart and electronic device sectors in different country contexts, such as Indonesia (Wijayaa et al., 2021). Hence, it implies that marketers should create a variety of hedonic content to encourage consumers to know and understand more about their brands. Because trendiness has been found to have a greater influence on generating brand awareness and brand image in comparison to other smart products industries in other country contexts such as Macau (Guan et al., 2022), marketers in Malaysia should provide personalised search features and the most up-to-date information highlighting their products and services on social media platforms, with the goal of establishing a strong brand image and attracting new consumers by providing a trendy feel. Furthermore, prior studies of similar smart devices demonstrated that SMMA was utilised as a platform for word of mouth, which was the most important driver of generating awareness (Ntumba and Budree, 2021). Given the study's similar findings, Malaysian portable tech-gadget and other smart digital device brands should focus on building social media view exchanges, chat rooms, or instant messaging to strengthen customers' perceptions in order to induce positive EWOM. Moreover, publicly discussing complaints about the products and services and the best-provided solutions on social media potentially increases EWOM. Finally, this study's results revealed INT as an insignificant factor in building brand equity. Therefore, marketers should be more cautiously utilising SMMAs to obtain market feedback to enhance consumers' brand interaction and product quality and encourage consumers to engage in two-way communication. As a whole, earlier research found that SMMAs seem more like an information source than a motivator of consumer behavior toward acquiring smart gadgets and other digital devices (Guan et al., 2022; Ntumba and Budree, 2021). In contrast, the current study revealed that the Malaysian portable tech-gadget industry has been heavily reliant on social media even during and after the COVID-19 disruption. This implies that industries producing similar technology products would be highly active in benefiting from SMMAs in order to boost their branding operations and achieve long-term profitability.

## 7 Conclusion

SMMAs are not new ideas in marketing, but the concept has continuously evolved and given marketers a fresh perspective on advanced marketing circumstances that are independent of time, location, or industry. The COVID-19 pandemic has caused critical implications to all aspects of human life. Despite this disruption, the economic sector has developed adequate strategies to efficiently conduct day-to-day operations following the rapid technology advancements in social media marketing. The current study analysed how SMMAs influence Malaysian consumers' brand equity, brand loyalty, and willingness to pay premium price for portable tech gadgets. This study's findings confirmed the reliance of Malaysian consumers on brands' SMMAs for tech gadget purchases. SMMAs enable marketers to directly connect with consumers and solicit consumers' feedback. Despite their efforts, SMMAs have not always been successful for many brands due to the inefficient utilisation. Only thorough knowledge and appropriate use of SMMAs can establish social media as an efficient marketing approach for businesses. Brands can build strong brand equity and brand loyalty via SMMAs by successfully fostering entertainment, customisation, trendiness, and favourable electronic word-of-mouth. The current study revealed that SMMAs may effectively develop a solid client base, boost consumer involvement, and build strong brand loyalty by offering tailored content and services. Delightful and honest brand participation through SMMAs may create considerable brand value and convince consumers to pay premium price for the brand.

Addressing a few identified limitations, this study presented several recommendations for future research. Firstly, as only youths were surveyed in this study, the current study's findings were not generalised to the overall population of Malaysian consumers of portable tech gadgets. More studies with a larger sample size and diverse populations may help generalise the proposed framework. Secondly, the current study applied a cross-sectional research design, which limited the controllability of unobserved heterogeneity and denied a solid foundation for demonstrating causation. It is recommended to conduct a longitudinal study to design, produce, and assess variable arrangements more effectively using long-term data. Finally, the current study focused on a particular sector and a few components of SMMAs. It is recommended for future research to concentrate on other industries and include other potential factors to gain comprehensive understanding on the effects of SMMAs.

## Declarations

### Author contribution statement

Chinnasamy Agamudainambhi Malarvizhi, Sreenivasan Jayashree and Tanvir Abir: Conceived and designed the experiments; Performed the experiments; Wrote the paper.

Abdullah Al Mamun and Farzana Naznen: Conceived and designed the experiments; Analyzed and interpreted the data; Contributed reagents, materials, analysis tools or data; Wrote the paper.

### Funding statement

This work was supported by Multimedia University.

### Data availability statement

Data included in article/supp. material/referenced in article.

### Declaration of interest's statement

The authors declare no conflict of interest.

### Additional information

No additional information is available for this paper.

## Appendix 1 Research Instrument

Entertainment

1. The social media of portable tech gadget brands is enjoyable
2. Using portable tech gadget brand's social media is fun
3. Content of portable tech gadget brand's social media seems interesting
4. It is exciting to use portable tech gadget brand's social media site
5. It is easy to kill time using portable tech gadget brand's social media site

Interaction

1. Information sharing is possible in the social media of portable tech gadget brands
2. Discussion and exchange of opinions is possible in the social media of portable tech gadget brands
3. Expression of opinions is easy in the social media of portable tech gadget brands
4. Portable tech gadget brand's social media interacts regularly with its followers and fans
5. It is easy to provide my opinion through portable tech gadget brand's social media.

Trendiness

1. The information shared in the social media of portable tech gadget brands is up to date
2. The use of social media by portable tech gadget brands is fashionable.
3. Contents visible in portable tech gadget brand's social media is the latest trend
4. Using portable tech gadget brand's social media is really trendy
5. Anything trendy is available on portable tech gadget brand's social media

Customization

1. The information that I need can be found in the social media of portable tech gadget brands
2. The social media of portable tech gadget brands provided the information that I needed
3. Portable tech gadget brand's social media makes purchase recommendations as per my requirements
4. I feel my needs are met by using portable tech gadget brand's social media
5. Portable tech gadget brand's social media facilitates personalized information search

Electronic Word of Month

1. I would recommend my friends to visit portable tech gadget brand's social media
2. I would encourage my friends and acquaintances to use portable tech gadget brand's social media
3. I would like to share my purchase experiences with friends and acquaintances on portable tech gadget brand's social media
4. I would like to pass information on brand, product, or services from Portable tech gadget brand's social media to my friends
5. I would like to upload content from Portable tech gadget brand's social media on my blog or micro blog

Brand Awareness

1. I am always aware of portable tech gadget brands
2. I am aware of the characteristics of portable tech gadget brands
3. I can always remember the logo of portable tech gadget brands
4. Characteristics of portable tech gadget brands come to my mind quickly
5. I can quickly recall the symbol of portable tech gadget brands

Brand Equity

1. I have an impressive memory regarding portable tech gadget brands
2. I am aware of the leading portable tech gadget companies
3. I am aware of the extensive experience of portable tech gadget companies
4. I am aware of the portable tech gadget brand, which is a representative of the portable tech gadget industry
5. I am aware of the customer-oriented portable tech gadget brands

Brand loyalty

1. I will suggest specific portable tech gadget brand to other consumers.
2. I would love to recommend specific portable tech gadget brand to my friends.
3. I intend to visit specific portable tech gadget brand's social media page again.
4. I consider myself to be loyal to one specific portable tech gadget brand
5. If the specific portable tech gadget brand's products are not available at the store, I would buy the same brand from some other store

Willingness to pay premium price

1. The price of specific portable tech gadget brand would have to increase quite a bit before I would switch to another brand.
2. I am willing to pay a higher price for specific portable tech gadget brand than for other brands.
3. I am willing to pay a lot more for specific portable tech gadget brand than for other brands.
4. I am willing to pay ___% more for X brand over other brands: 0%, 5%, 10%, 15%, 20% and more

## Appendix ASupplementary data

DATA - SMMA and Portable Tech Gadgets.csv DATA - SMMA and Portable Tech Gadgets.csv
